# Paroxysmal dyspnoea in asthma: Wheeze, ILO or dysfunctional breathing?

**DOI:** 10.3389/falgy.2022.1054791

**Published:** 2022-11-17

**Authors:** A. L. Crawford, J. D. Blakey, K. Baumwol

**Affiliations:** ^1^ Department of Respiratory Medicine, and Speech Pathology, Sir Charles Gairdner Hospital, WA, Perth, Australia; ^2^ Medical School, Curtin University Medical School, WA, Perth, Australia; ^3^ Internal Medicine, University of Western Australia, WA, Perth, Australia

**Keywords:** dyspnoea, asthma, inducible laryngeal obstruction, vocal cord dysfunction, continuous laryngoscopy in exercise, dysfunction breathing, hyperventilation syndrome

## Abstract

Paroxysms of dyspnoea in the general population are commonly reported and are frequently assumed to be asthma-related, especially if this diagnostic label has been previously applied. Often, this is not the case. Inducible Laryngeal Obstruction (ILO) and Dysfunctional Breathing (DB) are common comorbid conditions that go unrecognised in many difficult-to-treat asthmatics. On average, these patients have a delay in diagnosis of almost 5 years. This delay, along with ineffective, inappropriate escalation of asthma therapy, frequent hospital presentations for uncontrolled symptoms, and even intensive care admissions, magnifies patient morbidity and poor quality of life. ILO and DB have similar presentations and triggers to asthma. Differentiating between them can be challenging, especially in centres that do not have access to multidisciplinary subspecialty asthma services. Objectively confirming the diagnosis can likewise be challenging as symptoms fluctuate, and gold-standard investigations require extensive experience. This mini-review will summarise the clinical features of ILO and DB, with particular focus in the context of individuals treated for asthma. This narrative review will define each condition, highlight poignant aspects of the history and describe elements of the diagnostic pathway to gain objective confirmation.

## Definition of terms

Inducible Laryngeal Obstruction: As per ERS/ELS statement 2017, ILO “… describes an inappropriate, transient, reversible narrowing of the larynx in response to external triggers”.

Dysfunctional Breathing: As per Boulding et al. (2016) “*Dysfunctional breathing is a term describing breathing disorders where chronic changes in breathing pattern result in dyspnoea and other symptoms in the absence or in excess of the magnitude of physiological respiratory or cardiac disease”.*

## Introduction

Breathlessness is a commonly reported issue, with a prevalence of over 20% in most high-income nations in population-based studies (1). Respiratory symptoms are also the most commonly documented reason for attendance in primary care (2). There are many potential factors influencing reported dyspnoea including respiratory, cardiac, neuromuscular, haematological and psychological disease, as well as obesity, pain and locomotor limitation. In countries where asthma is highly prevalent such as the United Kingdom or Australia (3), patients with symptoms suggestive of asthma—such as episodic dyspnoea—are often commenced on therapy prior to, or even without, objective investigation (4, 5). Assuming breathlessness is due to asthma is particularly likely if this diagnostic label has previously been applied to an individual, even if tests of airflow obstruction are normal. Approximately one third of patients diagnosed with asthma in the primary care setting have no evidence of airway dysfunction on pulmonary function or methacholine bronchial challenge testing (6). International guidelines emphasize that non-asthmatic conditions are misdiagnosed as asthma in up to 30% of cases (7–9). This often leads to inappropriate commencement or over-prescription of asthma therapy, with the potential for drug-related adverse effects, particularly from the use of oral corticosteroids for uncontrolled symptoms. There is a clear dose-dependent relationship between cumulative systemic corticosteroid use and all-cause adverse outcomes (10) and higher cumulative doses of systemic corticosteroids are associated with increased healthcare resource utilisation (11). Concurrently, the lack of accurate diagnosis may result in ongoing physical and psychological morbidity as appropriate intervention is delayed (12).

Several conditions contributing to dyspnoea are more frequent in those with asthma than the general population (13), some of which are relatively readily identified such as obesity and coronary artery disease. It can be challenging however, to differentiate asthma symptoms from those caused by dysfunctional breathing pattern disorder (DB) and inducible laryngeal obstruction (ILO). These conditions appear comorbidly with asthma relatively commonly, with described prevalence in asthmatics ranging from 19 to 38% for ILO (14, 15), and 29% for DB (16).

A structured multidisciplinary assessment is therefore recommended in difficult-to-treat asthma (17), as identifying and managing comorbidities such as DB and ILO can be challenging. Using such an approach is associated with better outcomes than standard care (18). However, multi-disciplinary subspecialty asthma clinics are relatively rare in many countries, and identifying DB and ILO is an especial challenge in a general Respiratory clinic or in primary care setting where these conditions are less frequently encountered, and the index of suspicion may be lower.

This mini-review will therefore summarise the clinical features of DB and ILO with an especial focus on their identification in the context of individuals treated for asthma. The structure of this narrative review will be to define each condition, highlight key aspects of the history, and describe elements of the diagnostic pathway for objective confirmation.

## Inducible laryngeal obstruction

ILO describes a group of related conditions whereby abnormal movements of laryngeal structures lead to airflow limitation in the middle airway. The increased respiratory effort required to overcome this airflow limitation is perceived as breathlessness, and is well-described as an asthma mimic or comorbidity (19).

### The history

ILO can occur in anyone, but disproportionately affects younger women; case series suggest more than three quarters of ILO patients are female and their mean age is approximately 40 years old (20, 21). Concerningly, it appears ILO patients have an average delay in diagnosis of almost 5 years. They are frequently incorrectly labelled as having refractory asthma, with the lack of effect of asthma therapies leading to increased healthcare resource utilization and emergency presentations from uncontrolled symptoms. It can be difficult to differentiate ILO and acute asthma given they share similar triggers and symptoms of cough, dyspnoea and wheeze. Moreover, ILO is more common in people with asthma (19).

**Figure 1:**
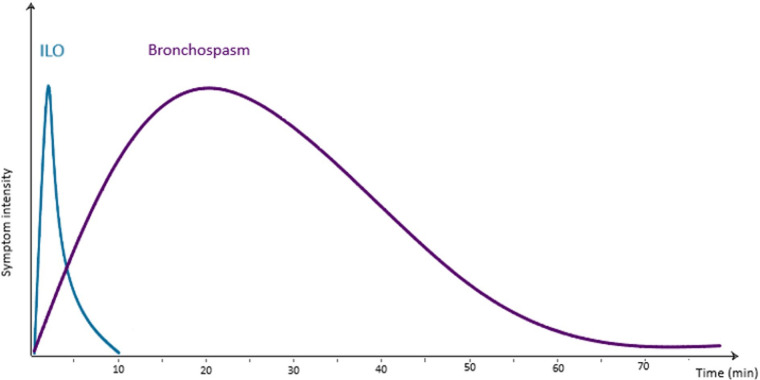
Approximate average timescale of symptoms of ILO and bronchospasm in relation to a triggering exposure.

As its name suggests, ILO is almost always “inducible”. This is to say it is often triggered by irritants and pungent smells such as cleaning products, perfumes, or cigarette smoke. It may also be set off by cold air, exercise or emotional stress. Triggers may vary between individuals but tend to be consistent within one patient. The triggering event usually leads to a rapid onset of symptoms within seconds to minutes. This contrasts with bronchospasm which almost always builds over many minutes (Figure 1). A typical history for ILO might involve someone noticing increasing symptoms as soon as they meet someone with liberally applied perfume. A typical history of asthma may involve symptoms increasing steadily over more than an hour whilst visiting someone who owns cats. As the issue is laryngeal, the patient's description of their “wheeze” will usually be helpful. Often an individual will describe their “wheeze” as an inspiratory or biphasic noise rather than no noise or a quiet end-expiratory polyphonic sound; this is a strong indicator toward a component of ILO. Pure expiratory laryngeal obstruction is described however is less common (22). The offset of symptoms is also usually relatively rapid in ILO, whereas asthma-related breathlessness tends to persist to some degree. Patients with ILO may become distressed at the lack of response to bronchodilators and therefore use repeated doses, resulting in a heightened sympathomimetic effect and thus increased muscle tension, anxiety, persistent symptoms and unscheduled healthcare attendance.

In the background history, an inciting event for the development of laryngeal sensitivity may well be uncovered, such as a respiratory tract infection or intubation for an unrelated procedure. Factors leading to the persistence of middle airway sensitivity are also common, such as post-nasal drip or gastro-oesophageal reflux. Asthma treatments such as inhaled steroids and excipients, and the low temperature of the plume from some pressured metered dose inhalers may sometimes also compound the problem.

Although people with ILO often also have a psychological diagnosis such as anxiety or depression, these conditions are highly prevalent in asthma (23) that the prior presence or absence of anxiety is not especially helpful diagnostically.

### Investigation

**Figure 2:**
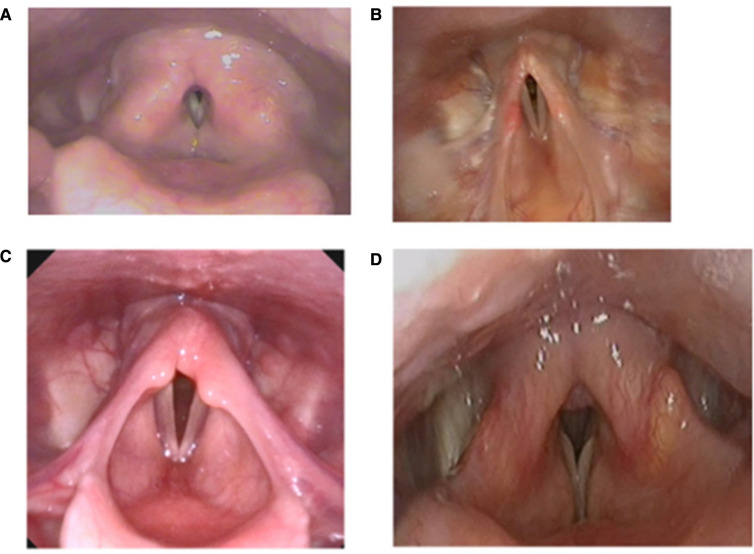
Laryngoscopy demonstrating (**A**) supraglottic constriction (**B**) aryepiglottic fold constriction, (**C**) shortened aryepiglottic folds and arytenoid prolapse at rest in an athlete with EILO, and (**D**) classic vocal cord dysfunction type ILO.

Confirming an ILO diagnosis usually requires the presence of compatible clinical features combined with direct laryngoscopic evidence, as per consensus international guidelines (19, 20). Flexible nasoendoscopy (FNE) should be undertaken *without* local anaesthetic, as diagnostic features may be missed. It is also imperative that efforts be made to trigger or reproduce the individual's symptoms in a laryngeal provocation assessment. Many individuals with confirmed ILO on provocation initially have an unremarkable examination whilst asymptomatic. The patient could be encouraged to replicate their breathing problem, and they should be exposed to a trigger they report such as a pungent perfume or rapid airflow. Examples of patterns of ILO are shown in Figures 2A–C, illustrating issues other than classical “Vocal Cord Dysfunction”. FNE is likewise crucial in excluding upper and middle airway structural disease, which can co-exist with ILO and may require further investigation and surgical review.

It's important to note that inspiratory flow-volume loops on spirometry often will not show classic variable extra-thoracic obstruction due to the rapid and fluctuating nature of ILO. Nevertheless, observing flow-volume loops for abnormalities can be informative in assessing for differentials.

Dynamic CT imaging using a standard protocol is an alternative means to diagnosis for some individuals, and is of increasing interest (24). This approach has the great advantage of not requiring a skilled endoscopist and is also not an aerosol generating procedure in the current context of the COVID pandemic. However, the difficulties in undertaking adequate laryngeal challenge in a scanner necessarily lead to some false negative results. Using FNE also allows biofeedback sessions with the patient able to see their laryngeal behaviour on screen. There is therefore a move to further develop and standardise patient pathways involving both CT and FNE to maximise accessibility and capacity, but also ensuring diagnostic accuracy and optimal therapy.

**Figure 3:**
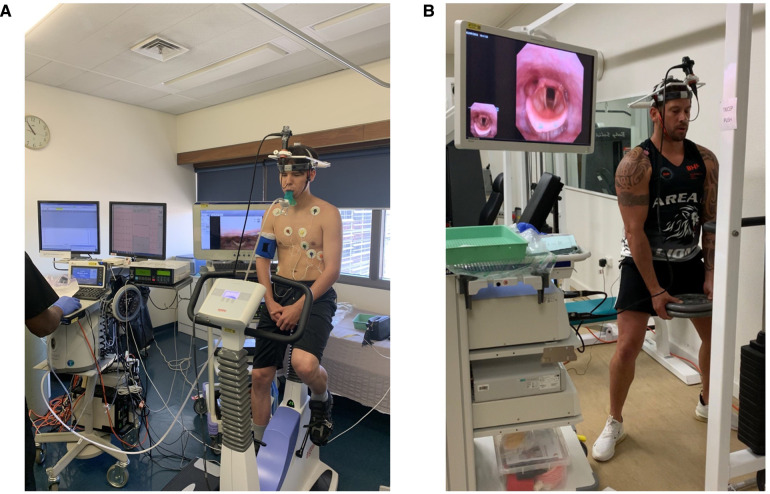
(**A**) patient prior to undergoing direct laryngoscopy with cardiopulmonary exercise testing. (**B**) Demonstrates a patient undergoing CLE whilst weightlifting. Both images are reproduced with the patients’ consent.

In the case of exercise-induced symptoms, it is important to note the type and duration of exercise. Bronchoconstriction generally occurs in prolonged or long-distance exercise due to mucosal dehydration. Exercise-induced ILO (EILO) typically occurs in exercises with rapid acceleration such as sprinting. EILO can occasionally be replicated by asking the patient to exert themselves then inserting the nasendoscope, however the diagnostic EILO images may be elusive due to rapid offset of abnormal laryngeal movements. As such, evidence of EILO should be sought by continuous laryngoscopy in exercise (CLE) as seen in Figure 3, preferably with the type of exercise that usually triggers an individual's symptoms (25).

When diagnosed, efforts should be made to provide the patient with management options. Several techniques have been developed to combat EILO including biofeedback and retraining, with promising results from a recent preliminary report of the novel Olin EILO biphasic inspiration (EILOBI) techniques (26).

## Dysfunctional breathing

Dysfunctional breathing (DB) describes a spectrum of abnormal breathing patterns that result in intermittent or chronic symptoms—predominantly dyspnoea. Symptoms occur in the absence of, or are disproportionate to, underlying cardiopulmonary disease. The essence of DB patterns is that they increase work of breathing through inefficient ventilation. They therefore contribute to an individual's sensation that their work of breathing is higher than they perceive it should be for a given exertion. This umbrella term is more appropriate than “hyperventilation syndrome”, which is only one of several types of DB that have been described. In our practice, we follow the nomenclature of Fowler and colleagues (16) and will discuss three subtypes that can be difficult to separate from asthma-related dyspnoea: forced abdominal expiration, erratic breathing, and thoraco-abdominal asynchrony (see Box 1). It's important to note that several DB patterns can co-exist in the same patient.

BOX 1 **| Three subtypes of dysfunctional breathing that may be mistaken for dyspnoea arising from asthma. Proposed nomenclature by Boulding R, Stacey R, Niven R and Fowler SJ, European Respiratory Review, 2016.**

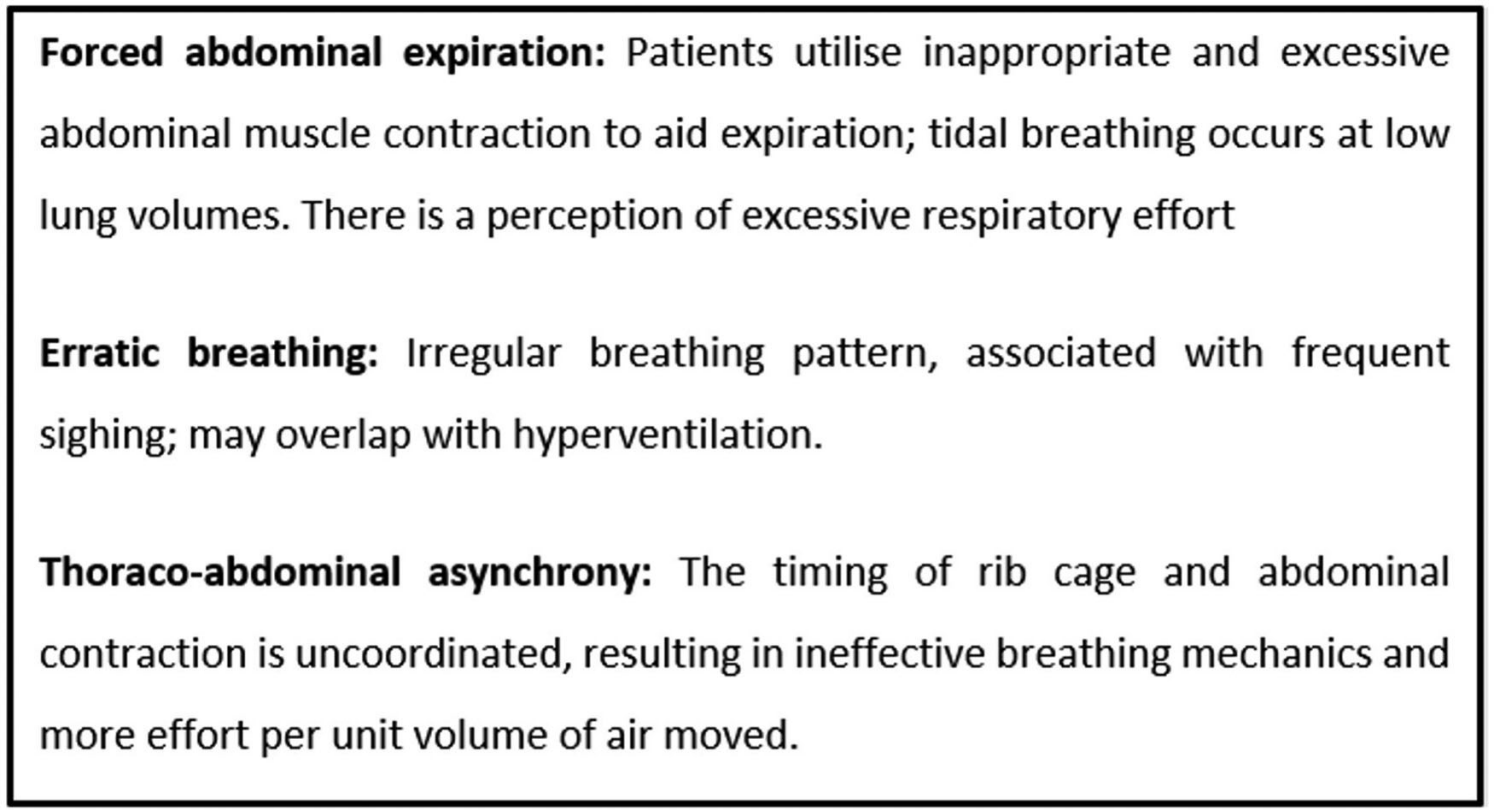

### The history

In the general population, the onset of dysfunctional breathing is usually with exertion, though it can also be a response to emotional stress, inhaled irritants, or manoeuvres such as those needed for spirometry. In patients with asthma, it can be triggered by the initial sensation of breathlessness due to non-severe bronchoconstriction or coughing. This can make identification of DB difficult and potentially lead to overtreatment of asthma. The prevalence of DB amongst asthma patients is uncertain, due to the lack of a gold standard for its diagnosis and the historic lack of consensus around classification. The prevalence of DB in difficult-to-treat asthma, however, is somewhat better understood with 30% experiencing DB as a major factor in their symptom complex (27).

DB in asthma is suggested by a relative lack of response to inhaled bronchodilators, and a persistence of episodes when regular treatment has been escalated. Patients may report minimal benefit from inhalers but a strong preference for nebulised therapy, or rather the several minutes sat resting with the latter underway. For most people with DB there is reported high work of breathing but relatively good exercise tolerance; patients may report they “soldier on” or “push through” when breathlessness occurs. In addition to dyspnoea, the classic features of hyperventilation may be present such as depersonalisation/derealisation, chest tightness, and peripheral paraesthesiae. The diagnosis in these cases is usually reached prior to specialist referral.

### Assessment

Questionnaires are frequently used to provide an assessment of respiratory complaints whilst the patient is in the waiting room. The Nijmegen questionnaire can sometimes be helpful in this context in terms of identifying hyperventilation, with reported specificity and sensitivity of 95% and 91% respectively (28). However, these values are much lower in practice as asthma and hyperventilation can produce similar symptoms. Crucially, many people with DB do not have straightforward hyperventilation that the questionnaire is intended to detect. An alternative patient reported outcome measure that may capture a broader range of DB is the Self Evaluation of Breathing Questionnaire (29), however this is also heavily influenced by comorbid respiratory disease from items such as “I can’t catch my breath” or “my breathing is heavy”.

Valuable information is often gained before the patient enters the clinic room. A suspicion of DB may arise when patients have difficulties with initial routine clinic tests. The breathing manoeuvre required to assess the fraction of expiratory nitric oxide (FeNO) requires a maintained steady expiration; this is challenging for those with types of DB other than hyperventilation. A discrepancy between reported limiting symptoms and reassuring spirometry also raises the index of suspicion. Review of the flow-loops rather than just the spirometric values can also be highly instructive: both an unusual shape of forced loop and a lack of reproducibility of individual efforts should raise the index of suspicion.

Observation of the patient and their breathing pattern is often the most instructive “test”. Tools such as the Breathing Pattern Assessment Tool (BPAT) are helpful in describing and categorising observation of a breathing pattern, facilitating discussion with colleagues and also monitoring of progress (30). We always watch the patient walking from the waiting area into the clinic room as this is often more informative than simply observing at rest. If this walk is short, or we have not observed them, we commonly ask the patient to walk with us along a corridor or potentially up the nearest stairs. Further highly instructive “observation” can be obtained by asking the patient to record a video of themselves when symptomatic.

Manual assessment of respiratory motion is a technique used to assess and quantify the distribution of breathing motion between the upper and lower parts of the rib cage, and the abdomen. While potentially useful, and widely employed by physiotherapists, relatively little research has been done to validate this technique. It requires a practitioner experienced not only in DB, but also in observing the breathing patterns of those with airflow obstruction. It remains unclear how much additional information this confers over observation.

### Specific investigations

As noted above, a review of simple clinic investigations such as spirometry may give a clue to abnormal breathing patterns. Discrepancy between observed and expected results is also of importance if individuals with asthma are assessed by both spirometry and forced or impulse oscillometry. Apparent restrictive or obstructive abnormalities in the forced manoeuvre may not be replicated in results from passive oscillometry testing.

Capnography will give an expected low-end tidal CO2 in some patients with hyperventilation syndrome, however its usefulness is limited as not all effortful breathing leads to sufficient increase in minute ventilation to reduce end tidal CO2.

**Figure 4:**
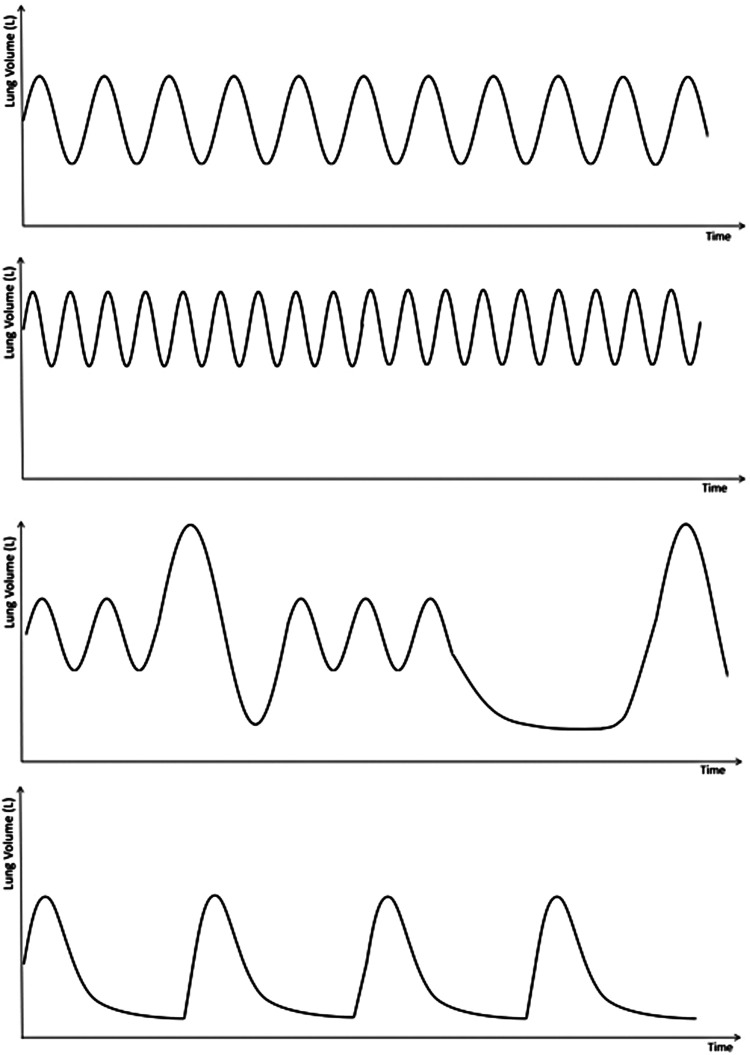
Representation of pneumotach readings, demonstrating: (**A**) normal tidal breathing; demonstrates a Normal breathing rate and rhythm. Note the placement of the breaths relative to the y axis—this demonstrates normal residual volume. (B) Hyperventilation: short, shallow, rapid breaths. Note the larger residual volume (**C**) periodic deep sighing with random breath hold: this graph shows normal tidal breathing interspersed with random deep sighs. Sighs are described as breaths with volumes 3 times the normal tidal volume. This graph also shows a random breath hold on expiration prior to a deep sigh. (**D**) Forced abdominal exhalation. Although not well described in the literature, this DB pattern results from excessive abdominal muscle use with longer expiratory phase and lower residual volume.

**Figure 5:**
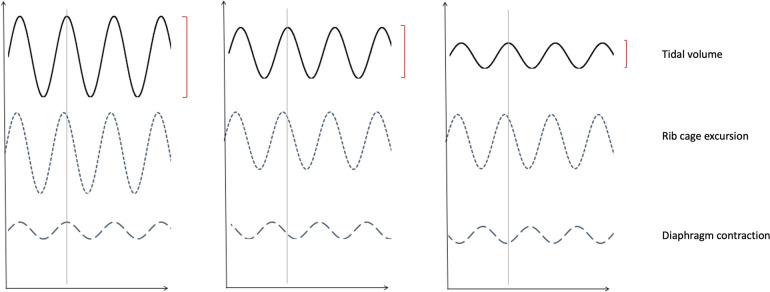
Impression of thoracic abdominal movements with respiratory cycle. (**A**) Demonstrates normal thoraco-abdominal movements with respiration, with (**B**) Illustrating partial dyssynchrony of diaphragm contraction and (**C**) complete asynchrony causing paradoxical diaphragmatic movement. Note the decreasing tidal volumes with progressive dyssynchrony.

A useful investigation is recording 1–2 min of tidal breathing *via* pneumotach (Figure 4) which can be followed by maximal expiration then inspiration. A comparison between healthy volunteer (a), and 3 patients with DB; hyperventilation (b) periodic deep sighing with random breath holds (c) and forced abdominal exhalation (d). Figure 5 illustrates the effect thoraco-abdominal asynchrony has on tidal volume through inefficient ventilation. This not only provides objective evidence of breathing pattern discrepancies between healthy and DB patients, but further discriminates between DB variants.

**Figure 6:**
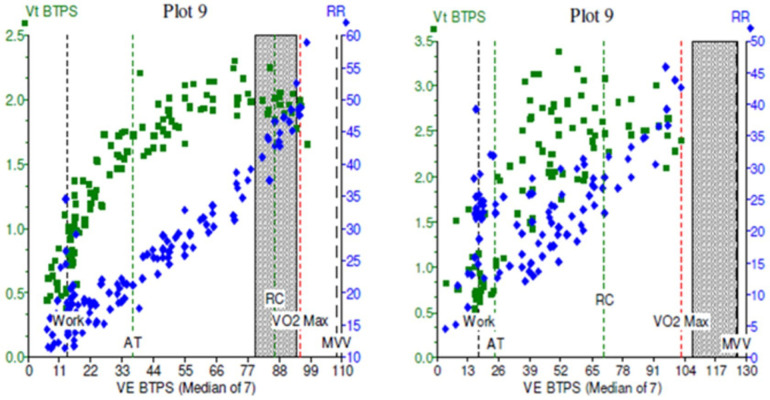
Cardiopulmonary exercise test (CPET) plots of minute ventilation (VE, *x* axis) against tidal volumes (Vt, *y* axis) in two patients. In (**A**), the green squares demonstrate a normal physiological response to incremental exercise, with increase Vt initially followed by increasing VE in a healthy subject. In (**B**), exercise was limited by dysfunctional breathing, with erratic, non-linear relationship between Vt and VE which becomes more noticeable at higher workloads.

Cardiopulmonary exercise testing (CPET) is considered the gold standard for evaluation of exertional dyspnoea and exercise intolerance (31). It is also an attractive method by which to identify the type and severity of DB. Observing parallel measurement of tidal volume (Vt), Ventilatory equivalents for CO2 (VE/VCO2), and for O2 (VE/VO2), and flow-volume loops gives an overview of breathing control and efficiency. Features of DB during CPET include an erratic pattern of both rate and tidal volume in response to exercise, and high ventilatory equivalents (Figure 6). In addition to characterising DB, it can also provide reassurance that other exercise-limiting factors such as cardiac, respiratory, metabolic, circulatory and muscular abnormalities are not at play (32, 33).

Optoelectric and Structured Light Plethysmography evaluate breathing mechanics by measuring the changes in volume of the chest wall, ribcage and abdomen. Such techniques appear potentially very useful in identifying and classifying DB, and in particular thoraco-abdominal asynchrony that may be missed by other approaches (34). These tools are, however, not in routine clinical practice and are currently primarily employed for research.

## Discussion

The term “asthma” is derived from a Greek stem meaning “to breathe hard”. For many years, people with episodic dyspnoea of a variety of aetiologies have been labelled as having asthma. Those that do not improve with standard inhaled therapy have been labelled “severe” or more correctly “difficult to treat” asthma (35). More recently, there have been substantial advances in our understanding of the treatable traits that contribute to an individual's asthma complex (36). This has been followed by an increasing uniformity in how these are measured, and in the consistency of terminology used to describe them. In difficult-to-treat asthma, identifying the comorbid “treatable traits” of ILO and DB ensures clinicians avoid potentially harmful overtreatment of asthma, particularly in terms of reducing the use of oral corticosteroids. The reduction in unscheduled healthcare utilisation has a positive effect on the patient's physical and psychological morbidity and reduces healthcare costs at a time when our healthcare systems are under great pressure (37).

Although ILO and DB may not immediately be straightforward to differentiate from asthma, time invested in taking a careful history and observing someone with symptoms is never wasted, with referral for investigations being preferable to inappropriate escalation of treatment. These actions are in keeping with general principles of practice and reflects the guideline-based encouragement to keep asthma diagnoses and treatments under regular review, with referral to specialist care when symptoms are persistent or discrepant from objective measures (17).

The prevalence and impact of these conditions is clearly significant, but progress is still required to adequately match clinical services to this need. Although pockets of excellence exist, not all individuals with these conditions have access to suitably qualified and experienced clinical staff, particularly working within a multi-disciplinary service (38).

In parallel with this need to augment capacity and specific medical, physiotherapy and speech pathology training, further research is needed. There remains great scope to improve our understanding of the most appropriate and efficient way to assess and treat individuals with DB and ILO.
